# Organizing Pneumonia as a side-effect of Na-valproate- a case report

**DOI:** 10.1192/j.eurpsy.2023.1621

**Published:** 2023-07-19

**Authors:** I. Rakos, D. Lucijanic

**Affiliations:** Psychiatry Clinic, University Hospital Dubrava, Zagreb, Croatia

## Abstract

**Introduction:**

Organizing pneumonia (OP) is a clinical, radiological and histological entity that is classified as an Interstitial Lung Disease. It can be either cryptogenic (of unknown cause) or secondary to a lung injury such as infection, drug toxicity, inhalation of a pathogen or toxic gas, gastroesophageal reflux, collagenosis, organ transplant, or radiotherapy (B.J. Roberton, D.M. Hansell. Organizing pneumonia: a kaleidoscope of concepts and morphologies. Eur Radiol, 21 (2011), pp. 2244-2254). We were called for a psychiatric consultation for a 50 years old male patient who presented to Emergency service of our hospital with symptoms of acute respiratory failure and bilateral pneumonia. This was his fourth hospital admission within two months with the same symptoms. In previous stays, he was given four different antiobiotics.

**Objectives:**

The objective of our psychiatric consult was to determine whether the clinical presentation of bilateral pneumonia could in fact be a side effect of one of the psychiatric drugs he was taking.

**Methods:**

We reviewed the patients prescribed medication and their side-effect profile. Additionally, the patient underwent a series of diagnostic tests, with the most important one being histology analysis of the biopsy samples.

**Results:**

Upon reviewing the available medical sources, we were able to find a few articles that link organizing pneumonia and use of Na-valproate (Nanau RM, Neuman MG. Adverse drug reactions induced by valproic acid. Clin Biochem. 2013;46:1323–1338). The said medication was discontinued and the patient started receiving corticostroids. After only a few days, his condition improved drastically and was discharged to home care.

**Conclusions:**

The mutual cooperation between internal medicine specialists and liaison psychiatrists is vital in cases like this when there is a psychiatric patient presenting with unspecific somatic symptoms or is responding poorly to standard treatment. We must sensitize the staff to the specifics of care for a psychiatric patient and at the same time provide him with adequate medical assistance.

**Disclosure of Interest:**

None Declared

